# Effects of Early Blood Pressure Lowering on Early and Long-Term Outcomes after Acute Stroke: An Updated Meta-Analysis

**DOI:** 10.1371/journal.pone.0097917

**Published:** 2014-05-22

**Authors:** Hongxuan Wang, Yamei Tang, Xiaoming Rong, Hui Li, Rui Pan, Yidong Wang, Ying Peng

**Affiliations:** Department of Neurology, Sun Yat-sen Memorial Hospital, Sun Yat-sen University, Guangzhou, Guangdong Province, China; INSERM U894, Centre de Psychiatrie et Neurosciences, Hopital Sainte-Anne and Université Paris 5, France

## Abstract

**Background:**

Hypertension is common after acute stroke onset. Previous studies showed controversial effects of early blood pressure (BP) lowering on stroke outcomes. The aim of this study is to assess the effects of early BP lowering on early and long-term outcomes after acute stroke.

**Methods:**

A meta-analysis was conducted with prospective randomized controlled trials assessing the effects of early BP lowering on early and long-term outcomes after acute stroke compared with placebo. Literature searching was performed in the databases from inception to December 2013. New evidence from recent trials were included. Outcomes were analyzed as early (within 30 days) and long-term (from 3 to 12 months) endpoints using summary estimates of relative risks (RR) and their 95% confidence intervals (CI) with the fixed-effect model or random-effect model.

**Results:**

Seventeen trials providing data from 13236 patients were included. Pooled results showed that early BP lowering after acute stroke onset was associated with more death within 30 days compared with placebo (RR: 1.34 and 95% CI: 1.02, 1.74, *p* = 0.03). However the results showed that early BP lowering had no evident effect on early neurological deterioration, early death within 7 days, long-term death, early and long-term dependency, early and long-term combination of death or dependency, long-term stroke recurrence, long-term myocardial infarction and long-term CVE.

**Conclusions:**

The new results lend no support to early BP lowering after acute stroke. Early BP lowering may increase death within 30 days after acute stroke.

## Introduction

Elevated blood pressure (BP) is common in acute phase of stroke onset; about more than 75% of patients with acute stroke have elevated blood pressure at presentation[1]–[3]. It may reflect untreated or uncontrolled hypertension before stroke, or it may relate to stress response, autonomic dysfunction or increased intracranial pressure after stroke onset[2], [3]. Observation studies have found that high blood pressure in acute stroke is associated with poor short-term and long-term outcomes[2], [4]–[6]. High blood pressure in acute stroke may be accompanied by higher risk of cerebral edema[7], hemorrhagic transformation of the infarct following thrombolysis in ischemic stroke[8] or expansion of the hematoma in hemorrhagic stroke[9]. In view of the potential risks of hypertension in acute stroke, epidemiologists suggest that high BP should be lowered. However, the high blood pressure usually decreases spontaneously in 4–10 days after stroke onset[10]. Observation studies have reported that low blood pressure in acute ischemic stroke is also associated with a poor prognosis[11], [12]. The mechanism may be that BP lowering may result in the reduction of cerebral blood flow because of the impaired auto-regulation after ischemic stroke, which leads to further ischemia in penumbra[13]. In consideration of similar mechanism, hypoperfusion in the perihematoma region of intracerebral hemorrhage may also occur after BP lowering. Hence, pathophysiologists argue that lowering BP should be of caution. Therefore, it is a clinical problem whether to lower high BP after acute stroke or not. The benefits of lowering the initial BP remain debated.

Previous studies about the effects of early BP lowering on the outcomes after acute stroke had given conflicting results. Several randomized controlled trials (RCTs) suggested that early BP lowering after acute stroke was safe[14]–[16]: The CHHIPS study showed early BP lowering in combination group of ischemic stroke and hemorrhagic stroke could improve long-term mortality[16]; the ACCESS study found early BP lowering in ischemic stroke could reduce recurrent vascular events[14]; and the INTERACT study reported early BP lowering in hemorrhagic stroke could reduce hematoma growth[15]. While the other RCTs found no evident benefit of early BP lowering after acute stroke[11], [17]. Therefore, previous cochrane meta-analyses had yielded neutral results[18], [19]. However, not all the trials included in previous analyses did achieve BP reduction in the intervention group, such as some studies with nimodipine in previous meta-analysis[19]. We argue that they could not lend direct support to the potential association between BP lowering and outcomes after acute stroke, and that they would also confound the association resulting in the obscure effects.

For recent years, some new studies with large sample focusing on the early BP lowering after acute stroke have still noted inconsistent results: the COSSACS study which enrolled patients with either ischemic stroke or hemorrhagic stroke, and the PRoFESS study which enrolled patients with ischemic stroke suggested safe but non-significant benefit of early BP intervention[20], [21], while the SCAST study which enrolled patients with either ischemic stroke or hemorrhagic stroke indicated a harmful effect of early BP lowering[22]. The INTERACT2 which enrolled patients with hemorrhagic stroke study indicated the trend to improve functional outcomes after intensive lowering of BP[23], but the CATIS study which enrolled patients with ischemic stroke[24] showed no difference between presence and absence of the antihypertensive medication in acute stroke. Considering these new evidence which was not included in previous meta-analyses[18], [19] and the aim to elucidate a new conclusion on the effects of early BP lowering after acute stroke, we update this meta-analysis. Additionally, we also assess the early neurological deterioration after BP lowering in acute stroke, which had not been discussed in previous meta-analyses.

## Methods

### Search strategy

We conducted the meta-analysis by reviewing published literature. We searched the following data sources: MELINE via Pubmed (from 1966 to December 2013), Science Citation Index (ISI Web of Science, from 1970 to December 2013), Cochrane Database of Systematic Reviews (CDSR) and the Cochrane Central Register of Controlled Trials (CENTRAL) with the search terms, key words or medical subject heading which included “blood pressure lowering”, “hypertension”, “anti-hypertension”, “antihypertensive” and “acute” and “stroke”, “cerebrovascular disease”, “cerebral infarction”, “brain infarction”, “cerebral ischemia”, “brain ischemia”, “cerebral hemorrhage”, “intracranial hemorrhage” and “randomized controlled trial”. The search was limited to trials in human beings and that published in English. In addition, we also screened the references of identified articles and previous reviews or meta-analyses on blood pressure lowering in acute stroke.

### Selection criteria and data extraction

We included the eligible trials which met the following criteria: (1) participants were aged 18 years or older with acute ischemic or hemorrhagic stroke; (2) study design was prospective randomized controlled trial; (3) intervention compared with placebo was initiated within 7 days of stroke onset; (4) intervention aimed to lower blood pressure or intervention achieved BP reduction; (5) one or more functional outcomes were reported, such as death or dependency. We excluded the studies with the patients of subarachnoid hemorrhage, studies without available full-text or relevant data, studies about ongoing trials and those written in languages other than English.

We extracted the data from each trial by reviewing published papers and even requesting detailed information from original authors if needed. Outcomes including early neurological deterioration (END), death, dependency, death or dependency, stroke recurrence, myocardial infarction and combined vascular events (CVE) were collected. We defined END and dependency as the original authors' definition in each study (END was defined as an increase in NIHSS of 4 or more points, or as a decrease in GCS score of 2 or more points in the CHHIPS, INTERACT and INTERACT2 study; dependency was defined as a mRS score of 3 or more points), and we stratified the outcomes to two phases as early outcome (within 30 days) and long-term outcome (3 to 12 months). We also defined CVE as the original authors' definitions (CVE included myocardial infarction, all types of stroke recurrence and peripheral vascular diseases).

Two authors (HW and XR) conducted the literature searches, data extraction and study assessment independently. Any disagreement in study selection or data collection was discussed by HW, XR and YT until agreement was reached. We also discussed the quality of trials such as randomization, blinded method, eligibility criteria and intention-to-treat analysis before they were finally included.

### Statistical analysis

Individual study relative risks (RR) and 95% confidence intervals (CI) were calculated from event numbers extracted from each trial before data pooling. In calculation of risk ratios, the total number of patients randomly assigned in each group was used as the denominator. Summary estimates of RR ratios were obtained with fixed-effect models or random-effect models. The percentage of variability across studies attributable to heterogeneity beyond chance was estimated with the I^2^ statistic. Potential heterogeneity was explored in estimates of treatment effect with univariate meta-regression and by comparing summary results obtained from the studies. A two-sided p value less than 0.05 was regarded as significant for all analyses. If the value of I^2^ was >50% in the analysis, the heterogeneity between studies was defined as significant, and the random-effect model was used for analyses. Otherwise, fixed-effect models was used. Funnel plots were used for analyses of bias. The Cochrane Collaboration's Review Manager software package (RevMan, Edition 5.2) was used for the meta-analyses.

## Results

### Summaries of included trials

**Figure 1:**
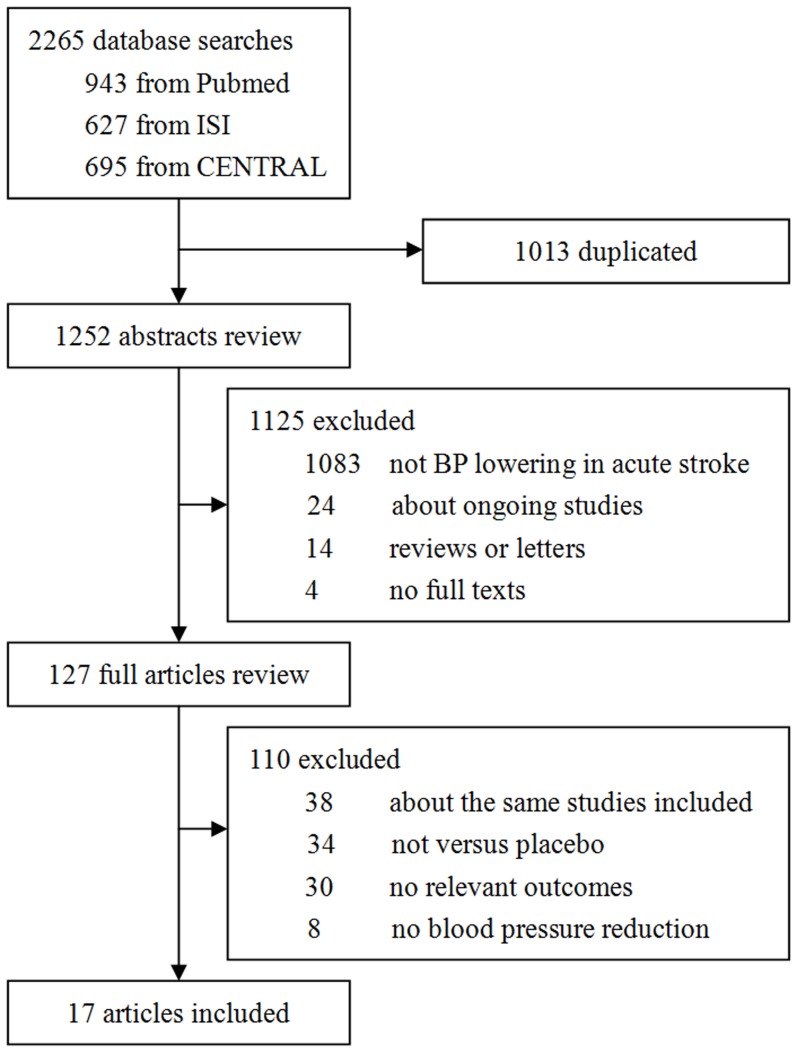
Identification process for included studies. The figure shows detailed information in the process of search, review, exclusion and inclusion of the potential articles.

The literature search and the review of previous meta-analyses or reviews finally yielded 17 studies which met the inclusion criteria (Figure 1). The characteristics of the included studies are summarized in Table 1.These studies included a total of 13236 patients from various countries such as UK, USA, Germany, Japan, China, and so on. The process of identifying the included articles is shown in Figure 1. All data included in this analysis were extracted from original papers only because of no additional data available from requested authors.

**Table 1:** Summary of included studies.

Study	Year	Country of origin	No. of patients	Male (%) (I; C)	Mean age (years) (I; C)	Stroke type	Inclusion of onset (hours)	Mean admission time (hours) (I; C)	Agent class	Intervention period (days)	Duration of follow-up (months)	Study scale	Outcome
ACCESS[14]	2003	Multicentre in Germany	339	51(50;52)	68;68	IS	36	29.9;29.7	ARB	7	12	BI	Death, stroke recurrence, MI
Bath *et al* [30]	2001	Single centre in UK	37	49(38;57)	76;72	IS+HS	120	105.6;93.6	GTN	12	3	RS	Death, dependency
BEST[29]	1988	Single centre in UK	302	54;49	70;69	IS+HS	48	23.4;25.3	BRB	21	6	Ordinal scale	Death, SAE
CATIS[24]	2013	Multicentre in China	4071	64(65;63)	62;62	IS	48	15.3;14.9	All kinds	7	3	NIHSS,mRS	Death, dependency
CHHIPS[16]	2009	Multicentre in UK	179	55(57;53)	74;74	IS+HS	36	19.8;17.4	ACEI/BRB	14	3	NIHSS, mRS	Neurological worsening, death, dependency
COSSACS[21]	2010	Multicentre in UK	763	56(55;56)	74;74	IS+HS	48	23.6;23.4	All kinds	14	6	NIHSS, mRS, BI	Death, dependency, stroke recurrence, MI
Eveson *et al* [26]	2007	Single centre in UK	40	63(44;77)	73;75	IS	24	18.0;20.0	ACEI	14	3	NIHSS, mRS, BI	Death, dependency
Hsu *et al* [28]	1987	Multicentre in USA	80	61(58;65)	63;65	IS	24	15.3;17.4	PGI_2_	3	1	Neurologic Grading Scale	Neurological worsening, death
INTERACT[15]	2008	Multicenter from Australia, China and South Korea	404	65(61;69)	63;62	HS	6	3.6;3.7	All kinds	7	3	NIHSS, GCS, mRS	Neurological worsening, death, dependency, stroke recurrence
INTERACT2[23]	2013	Multicentre from Australia, China and South Korea	2839	63(64;62)	63;64	HS	6	3.7;3.7	All kinds	7	3	NIHSS, mRS	Death, dependency, SAE
INWEST[11]	2000	Multicentre from Sweden	295	45	72	IS	24	/	CCB (iv.)	21	6	BI	Death, dependency
Kaste *et al* [27]	1994	Multicentre in Finland	350	67(69;65)	57;58	IS	48	/	CCB (po.)	21	12	Sum score, RS	Death, dependency
Nakamura *et al* [25]	2010	Single centre in Japan	40	83(81;85)	62;81	IS	72	45;41	ARB/ACEI	14	0.5	NIHSS, mRS	Neurological worsening, death, stroke recurrence, MI, SAE
PRoFESS[20]	2009	Multicentre in 35 countries	1360	65(65;65)	67;67	IS	72	57.6;57.6	ARB	90	3	NIHSS, mRS	Death, dependency, stroke recurrence, MI, SAE
Rashid *et al* [17]	2003	Single centre in UK	90	46(47;43)	71;74	IS+HS	72	51.0;49.5	GTN	10	3	SNSS, mRS, BI	Neurological worsening, death, dependency
SCAST[22]	2011	Multicentre in 9 north European countries	2029	58(60;56)	71;71	IS+HS	30	17.6;17.9	ARB	7	6	SSS, mRS, BI	Death, dependency, stroke recurrence, MI
Willmot *et al* [31]	2006	Single centre in UK	18	28(17;50)	69;70	IS+HS	120	79;77	GTN	7	3	SSS, mRS	Death

I: intervention; C: control. ACEI, angiotensin converting enzyme inhibitors; ARB, angiotensin receptor blockers; BRA, beta receptor antagonists; CCB, calcium channel blockers; GTN, glyceryl trinitrate. BI, Barthel Index; GCS, Glasgow Coma Scale; NIHSS, NIH Stroke Scale; RS, Rankin Scale; SSS, Scandinavian Stroke Scale.

The trials had a sample size ranging from 18 to 4071 patients, of which eleven were multi-centre randomized controlled trials (RCT) and the others were single centre RCT. Eight trials[11], [14], [20], [24]–[28] included patients with acute ischemic stroke (IS) only and two trials[15], [23] included patients with acute hemorrhagic stroke (HS) only, while seven trials[16], [17], [21], [22], [29]–[31] included patients with either type of stoke except subarachnoid hemorrhage. Most trials had recruited more male than female. Patients had a mean age ranging from 57 to 76 years old. Patients were recruited into trials within 6 to 120 hours from stroke onset and the mean time to randomize to intervention ranged from 3.6 to 105.6 hours. The trials studied the effect of one or more kinds of antihypertensive agents including the following types: angiotensin converting enzyme inhibitors (ACEI), angiotensin receptor blockers (ARB), beta receptor antagonists (BRA), calcium channel blockers (CCB), glyceryl trinitrate (GTN) and prostacyclin. Intervention period ranged from 3 to 21 days except that the intervention period of the PRoFESS study was 3 months. Follow-up durations ranged from 14 days to 12 months (Table 1).

### Early neurological deterioration

**Figure 2:**
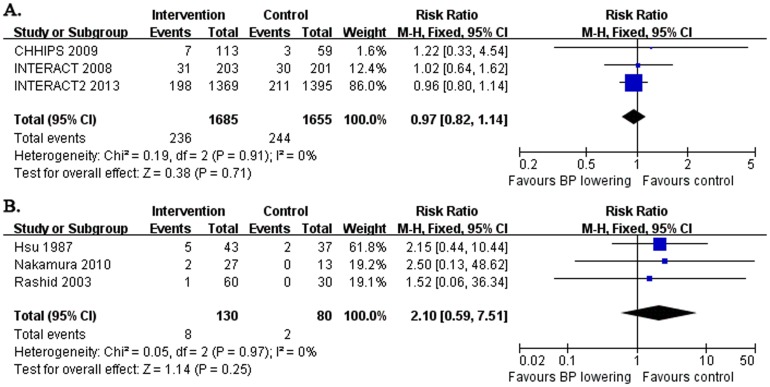
Early neurological deterioration after acute stroke. The figure shows meta-analysis of early BP lowering on early neurological deterioration within 72 hours (A.) or within 14 days (B.) after acute stroke. Risk ratios (RR) and their 95% confidence intervals (CI) were estimated event rates of intervention (BP lowering) compared with control (placebo). Overall effects were tested by Z tests and the heterogeneity of between-studies was tested by χ^2^ test and measured as the value of *I^2^*.

Six trials mentioned early neurological deterioration (END) after early BP lowering in acute stroke (Table 1, Figure 2). Of them, three studies reported END within 72 hours after intervention assigned (Figure 2A), which enrolled the patients within 6 to 36 hours after stroke onset. And the other three studies recorded END within 14 days (Figure 2B), which enrolled the patients within 24 to 72 hours after stroke onset. The pooled result showed that early BP lowering after acute stroke yielded no effect on END within 72 hours (RR: 0.97 and 95% CI: 0.82, 1.14) or within 14 days (RR: 2.10 and 95% CI: 0.59, 7.51). And the heterogeneity between studies was not evident (both *I^2^* = 0%, *p*>0.05).

### Early and long-term death

**Figure 3:**
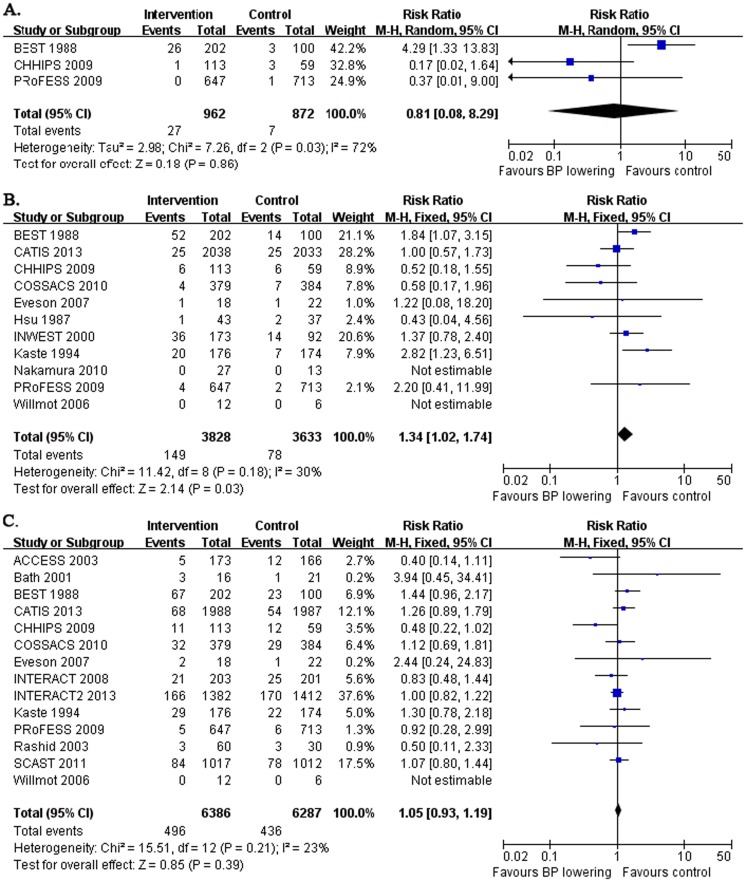
Early and long-term death after acute stroke. The figure shows meta-analysis of early BP lowering on early death within 7 days (A.) or within 30 days (B.) and long-term death from 3 to 12 months (C.) after acute stroke. Risk ratios (RR) and their 95% confidence intervals (CI) were estimated event rates of intervention (BP lowering) compared with control (placebo). Overall effects were tested by Z tests and the heterogeneity of between-studies was tested by χ^2^ test and measured as the value of *I^2^*.

Three studies recorded early death within 7 days after early BP lowering in acute stroke, which enrolled the patients within 36 to 72 hours after stroke onset (Table 1, Figure 3A). Overall, no significant effect of early death after early BP lowering within 7 days was showed (RR: 0.81 and 95% CI: 0.08, 8.29, Figure 3A). Although the heterogeneity between studies was noted (*I^2^* = 72%, *p* = 0.03) and it was due to the discrepant data from the BEST trials, however, there was no clinical reason to exclude it from the analysis and no other potential subgroup analysis to eliminate the heterogeneity.

Eleven studies followed up early death for 14 to 30 days after early BP lowering in acute stroke, which enrolled the patients within 24 to 120 hours after stroke onset (Table 1, Figure 3B). Pooled results indicated the significant harmful effect that early BP lowering after acute stroke onset was associated with more death within 30 days (RR: 1.34 and 95% CI: 1.02, 1.74, *p* = 0.03, without significant heterogeneity between studies). Considering the broad span of admission time after stroke onset in the trials included in the analysis, we narrowed the inclusion criteria for the remaining 10 trials which enrolled the patients within 24 to 72 hours after stroke onset (excluding the Willmot's study), and the pooled results were not changed and the adverse effect of early BP lowering on death within 30 days was still evident (data not shown).

Fourteen studies followed up long-term death from 3 to 12 months after acute stroke, which enrolled the patients within 6 to 120 hours after stroke onset (Table 1, Figure 3C). No effect of early BP lowering was shown (RR: 1.05 and 95% CI: 0.93, 1.19). If we re-evaluated the pooled results by narrowing the inclusion criteria for the remaining 12 trials with the admission time within 6 to 72 hours after stroke onset, the result was not changed (data not shown).

### Early and long-term dependency, combination of death or dependency

**Figure 4:**
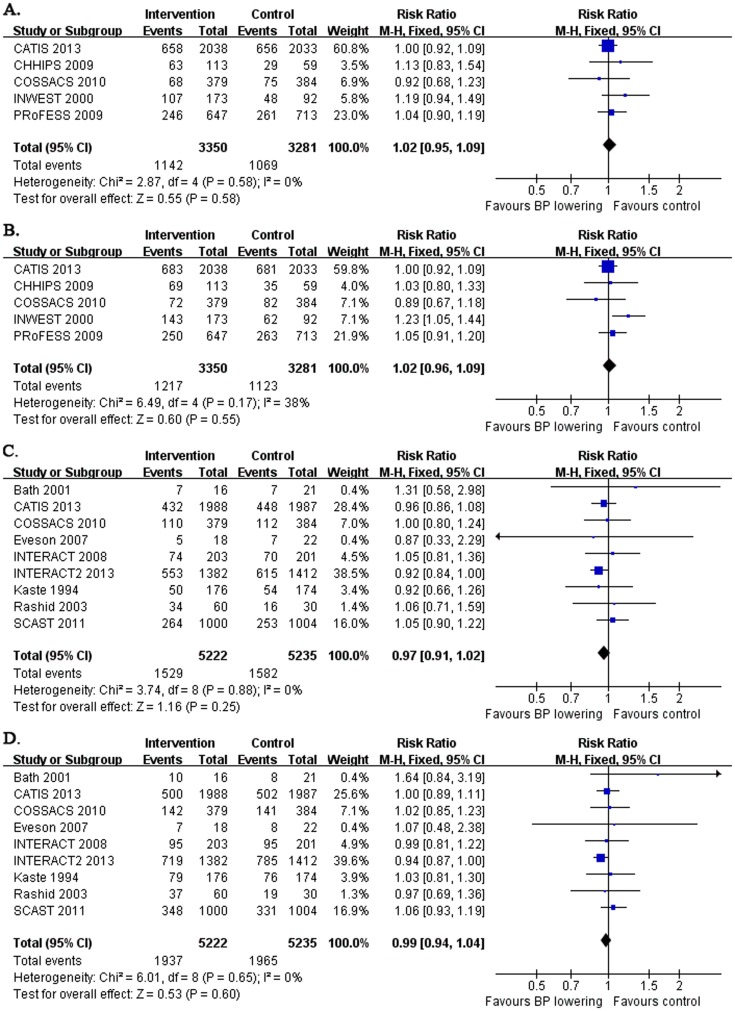
Early and long-term dependency, the combination of death or dependency after acute stroke. The figure shows meta-analysis of early BP lowering on early dependency (A.), early death or dependency (B.), long-term dependency from 3 to 12 months (C.) and long-term death or dependency from 3 to 12 months (D.) after acute stroke. Risk ratios (RR) and their 95% confidence intervals (CI) were estimated event rates of intervention (BP lowering) compared with control (placebo). Overall effects were tested by Z tests and the heterogeneity of between-studies was tested by χ^2^ test and measured as the value of *I^2^*.

Five studies reported early dependency and early death or dependency, which enrolled the patients within 24 to 72 hours after stroke onset (Table 1, Figure 4A, 4B). Pooled results showed that early BP lowering had no effect on early dependency (RR: 1.02 and 95% CI: 0.95, 1.09; without significant heterogeneity between studies) or early death or dependency (RR: 1.02 and 95% CI: 0.96, 1.09; without significant heterogeneity between studies).

Nine studies reported long-term dependency and long-term death or dependency, which enrolled the patients within 6 to 120 hours after stroke onset (Table 1, Figure 4C, 4D). Pooled results showed that early BP lowering had no effect on long-term dependency (RR: 0.97 and 95% CI: 0.91, 1.02; without significant heterogeneity between studies) and long-term death or dependency (RR: 0.99 and 95% CI: 0.94, 1.04; without significant heterogeneity between studies). Further analysis by narrowing the inclusion criteria for the remaining trials (excluding the Bath's study) with the admission time within 6 to 72 hours after stroke onset did not changed the neutral results (data not shown).

### Long-term vascular events

**Figure 5:**
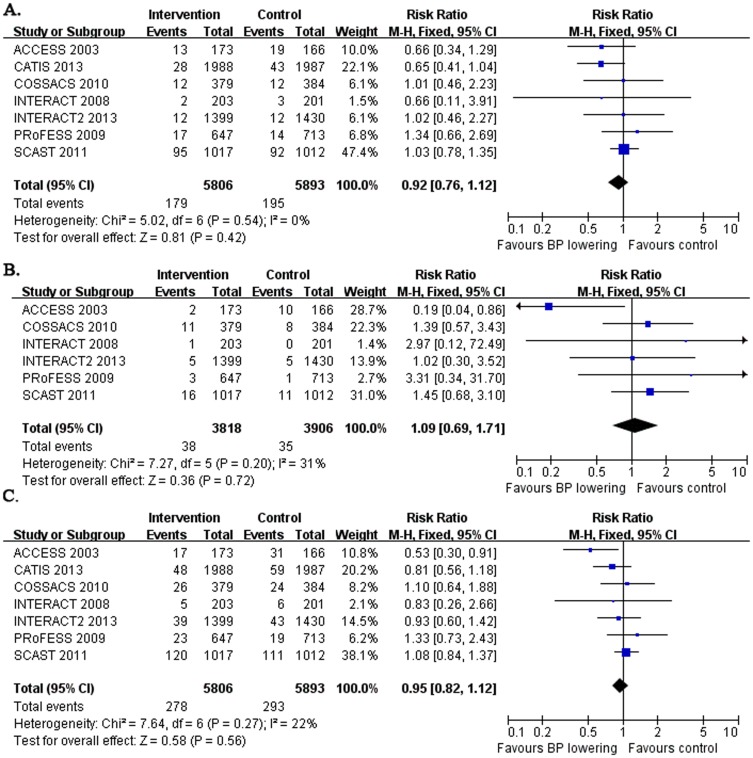
Long-term vascular events. The figure shows meta-analysis of early BP lowering on long-term stroke recurrence (A.), long-term myocardial infarction (B.) and long-term combined vascular events (CVE) from 3 to 12 months (C.) after acute stroke. Risk ratios (RR) and their 95% confidence intervals (CI) were estimated event rates of intervention (BP lowering) compared with control (placebo). Overall effects were tested by Z tests and the heterogeneity of between-studies was tested by χ^2^ test and measured as the value of *I^2^*.

Seven studies considered the long-term effect of early BP lowering on stroke recurrence and combined vascular events (CVE) (Figure 5A, 5C) and six studies reported the long-term effect of early BP lowering on myocardial infarction (MI) (Figure 5B) from 3 to 12 months after acute stroke onset, which enrolled the patients within 6 to 72 hours after stroke onset. Pooled data showed that early BP lowering had no effect on long-term stroke recurrence (RR: 0.92 and 95% CI: 0.76, 1.12; with no evidence of heterogeneity between studies), long-term MI (RR: 1.09 and 95% CI: 0.69, 1.71; with no evidence of heterogeneity between studies) and CVE (RR: 0.95 and 95% CI: 0.82, 1.12; with no evidence of heterogeneity between studies).

We used funnel plots to evaluated the publication bias. It showed no evident publication bias in the majority of outcomes such as END within 72 hours, END within 14 days, early death within 30 days, long-term death, early and long-term dependency, early and long-term combination of death or dependency and long-term stroke recurrence (data and plots not shown). Asymmetric funnel plots were shown in few results such as early death within 7 days, long-term MI and long-term CVE, which just only yielded neutral results in our analysis (data not shown).

## Discussion

Whether to lower high blood pressure early after acute stroke or not still remains debated. One cochrane meta-analysis failed to analyze the effect of BP lowering on stroke because the author could not obtain enough data[32]. The other two meta-analyses showed no significant effects of early BP lowering on outcomes after acute stroke[18], [19]. However, they did not include recent new trials and they did not assess the early neurological deterioration after BP lowering. Furthermore, they included some trials in which the intervention groups did not achieve BP reduction during the study[19]. We doubt that the results were confounded. In this study, we review the evidence from several new trials with large sample such as PRoFESS[20], COSSACS[21], SCAST[22], INTERACT2[23] and CATIS[24] which have not been included in previous meta-analyses[18], [19]. We also assess the early neurological deterioration after BP lowering in acute stroke, which has not been discussed in previous meta-analysis. This updated meta-analysis including overall 13236 patients from 17 trials notes the evidence that early BP lowering in acute stroke increases death within 30 days. However there is still no evident effect of early BP lowering after acute stroke on early neurological deterioration, early death within 7 days, long-term death, early and long-term dependency, early and long-term death or dependency, long-term stroke recurrence, long-term myocardial infarction and long-term CVE.

This analysis assesses early neurological deterioration after early BP lowering in acute stroke, which has not been discussed in previous reviews or meta-analyses. How ever, limited trials reported relevant data so that no certain effect can be found. So far, we could not draw any conclusion that early BP lowering after acute stroke is safe enough. More RCTs concerning BP lowering on early neurological deterioration within 72 hours are needed.

Considering potential confounding effect in previous meta-analyses because they included several trials without achieving BP reduction indeed (e.g. the Norris's study[33] and the VENUS study[34] in the Geeganage's analysis[19]). We doubt that probable harmful effects of BP lowering in these trials was attenuated because of no significant BP changes, so we just include the trials aiming to or achieving BP reduction in the intervention groups. The new result yields evident harmful effect of early BP lowering on early death within 30 days.

Because admission time after stroke onset may also be critical in the effect of BP lowering, it is best to make further subgroup analyses according to different admission time to show potential different effects of BP lowering on stroke outcomes. However, nearly no relevant data could be extracted or provided from the origin studies for further subgroup analyses. For it seems non-reasonable to combine the data from the patients enrolled within 6 hours after stroke onset with those from the patients enrolled within 120 hours after stroke onset[18], [19], therefore we further evaluated the pooled results by narrowing the inclusion criteria of admission time. In the included trials, only 2 trials (the Bath's study and the Willmot's study) enrolled the patients of wide admission time span up to 5 days (120 hours) after stroke onset, all the other studies enrolled the patients within 6 to 72 hours after stroke onset. If the 2 trials were excluded for a stricter inclusion criteria of admission time, similarly, the adverse effects on death within 30 days was still significant and the other neutral results would not be changed (data not shown). However, more studies concerning the timing of the intervention and different effects on outcomes after stroke onset are extremely required in future. In addition, we also included the studies of which admission time was within 72 hours (such as the PRoFESS study), because the studies did include part of patients who were enrolled within 48 hours after stroke onset. If we restricted a more stricter inclusion criteria of admission time which was from 6 hours to 48 hours, three more studies (the Nakamura's study, Rashid's study and the PRoFESS study) would be excluded but the pooled results of adverse effects on death within 30 days was still significant and the other neutral results would not be changed (data not shown).

Although the PRoFESS study was designed to focus on the effects of BP-lowering and antiplatelet strategies on secondary stroke prevention which enrolled totally 20332 patients, however, about 7% (1360) of the patients were enrolled within 72 hours after stroke onset and the intervention or placebo was assigned in the acute phase of stroke. Further more, the PRoFESS study was a 2×2 factorial design trial, which also had sufficient statistical power to detect the independent effect of BP lowering on outcomes after stroke. Therefore, the PRoFESS subgroup analysis can reasonably represent a single trial to test the effects of BP lowering on outcomes after acute stroke. On the other hand, the period of intervention in the PRoFESS study was longer (90 days) than other studies because of its initial aim for the secondary stroke prevention. We can not exclude the confounding effects due to mixed effects of short-term and long-term BP lowering. In our analysis, we found that the data from PRoFESS study showed different trends of effects of BP lowering on early death within 30 days after acute stroke (Figure 3B) and long-term death after 3 months after acute stroke (Figure 3C). On the basis of this results, we assumed that the PRoFESS study did show some effect of BP lowering after acute stroke and we could not exclude this trial from our analysis.

This updated meta-analysis differentiate from previous studies[19] with two more important aspects other than adding new data from recent trials or analyzing new outcome. Firstly, we just include the trials aiming to or achieving BP reduction in the intervention groups. In addition, we further evaluate the results by narrowing admission time span with a stricter inclusion criteria of the trials, and still find similar pooled results.

However,there are still several limitations in this analysis. Firstly, we could not obtain enough the data from the original authors, so several pooled outcomes could not be evaluated with sufficient studies and data, and it may affect our results more or less. Secondly, we could not make further subgroup analysis between ischemic stroke and hemorrhagic stroke because of the unavailable data of individual patients in each trial. But we did a crude subgroup analysis by different types of stroke with insufficient data and found that early BP lowering had a trend to increase early death within 30 days in ischemic stroke, which was similar to the combined result, however, no trend could be found in hemorrhagic stroke (data and figure not shown). Thirdly, we also could not make further subgroup analysis of the effects between different timing of intervention for the above reason. Finally, we could not assess the effects of different types of antihypertensive agents because of limited data available for detailed subgroup analyses. Therefore, more studies concerning the above problems are required in future and they will help to clarify the specific question more clearly on BP lowering after acute stroke, such as whether or when to intervene and which agent to use.

In conclusion, this updated meta-analysis with new evidence lend no support to early blood pressure lowering after acute stroke. It indicates that early BP lowering significantly increases death within 30 days after acute stroke.

## Supporting Information

Checklist S1 **PRISMA 2009 Checklist.**
(DOC)
